# Application of the Pipeline Embolization Device for Giant Vertebrobasilar Dissecting Aneurysms in Pediatric Patients

**DOI:** 10.3389/fneur.2019.00179

**Published:** 2019-03-11

**Authors:** Jiejun Wang, Yisen Zhang, Ming Lv, Xinjian Yang, Zhongbin Tian, Jian Liu, Peng Liu, Zefeng Miao, Luqiong Jia, Junfan Chen, Xinghuan Ding, Ying Zhang, Wei Zhu, Wenqiang Li, Kun Wang, Zhongxiao Wang

**Affiliations:** ^1^Beijing Neurosurgical Institute, Beijing Tiantan Hospital, Capital Medical University, Beijing, China; ^2^Heping Hospital Affiliated to Changzhi Medical College, Changzhi, China

**Keywords:** pediatric, dissecting, giant, vertebrobasilar, pipeline

## Abstract

**Objective:** To evaluate the feasibility and effectiveness of the pipeline embolization device (PED) for the treatment of pediatric giant vertebrobasilar dissecting aneurysms (VBDAs).

**Methods:** We retrospectively reviewed our institutional clinical database and identified 2,706 patients who presented with a diagnosis of intracranial aneurysms from January 2016 to June 2018. Among this group, 153 patients were diagnosed with VBDAs, and 54 of these patients underwent PED therapy. The PED technique was used in four patients who were 18 years old or younger at the time of presentation (two males, two females; mean age 9.25 years; age range 8–11 years).

**Results:** All four included pediatric patients were managed with the PED. One patient (25%) was treated with the PED alone, while three (75%) were treated with the PED and coils. One patient died from brainstem infarction or compression of the brainstem, while follow-up of the other three patients revealed favorable outcomes. The mass effect was reduced in cases 1, 2, and 3 on follow-up MRI performed 6 months after the PED procedure.

**Conclusions:** PEDs could be feasible in the treatment of pediatric giant VBDAs. However, the safety and efficacy of this method have not been clarified in this special pediatric population, and long-term follow-up is still necessary.

## Introduction

Pediatric intracranial aneurysms are exceedingly rare, accounting for <5% of all intracranial aneurysms, and intracranial dissecting aneurysms are even rarer (1, 2). Intracranial dissection mostly involves the vertebrobasilar circulation (3). Treatment of intracranial artery dissections is empirical, as there is an absence of data from randomized controlled trials. Patients with intracranial artery dissection with or without subarachnoid hemorrhage are usually treated with surgical or endovascular procedures (4). For pediatric patients with intracranial artery dissection, parental biases toward non-craniotomy therapy must be thoroughly addressed before the ultimate selection of a treatment strategy (5). Thus, endovascular procedures have become the first choice for the treatment of pediatric intracranial dissecting aneurysms. An endovascular procedure that has gained increasing popularity is the pipeline embolization device (PED). However, the safety and effectiveness of the PED for pediatric vertebrobasilar dissecting aneurysms (VBDAs) have not yet been clarified, as most of the literature on this topic is composed of case reports. In the present paper, we present our early experience in using the PED to treat VBDAs in four pediatric patients. The purpose of the present study was to evaluate the procedural feasibility and effectiveness of using the PED to treat pediatric VBDAs.

## Materials and Methods

### Patient Population

The present retrospective study was approved by the ethics committee of our institution. Written informed consent for study inclusion was obtained from the parents of all included patients. Between January 2016 and June 2018, 2,706 patients were referred to our department for endovascular treatment of an intracranial aneurysm. Among this group, 153 patients were diagnosed with VBDAs, and 54 patients agreed to undergo PED therapy. Of these 54 patients, four were 18 years old or younger at the time of presentation (two males, and two females; mean age 9.25 years; age range 8–11 years). None of these four cases involved traumatic aneurysms and/or collagen vascular disorders. Patient demographics and clinical information collected from the medical records are shown in Table 1.

**Table 1:** Patient demographics and clinical information on admission.

**Case**	**Age (years)**	**Presenting symptoms**	**Lesion site**	**Treatment modalities**	**Treatment outcome of aneurysms**
1	10–12	Headache and left-sided occipital pain	LVA	Pipeline+Coils	Incomplete occlusion
2	8–10	Intermittent headache and diplopia	VBJ	RVA-Pipeline+Coils; LVA-Balloon occlusion	Partial occlusion
3	8–10	Headache and dysphagia	VBJ	LVA-Pipeline+Coils; RVA-PAO	Partial occlusion
4	10–12	Headache and vertigo	BA	Pipeline	Contrast stasis

*LVA, left vertebral artery; RVA, right vertebral artery; BA, basilar artery; VBJ, vertebrobasilar junction*.

### Endovascular Procedure

We selected endovascular treatment as the first choice for these pediatric patients after comprehensive discussion within a comparatively full-scale group of researchers including pediatric experts, neurologists, and radiologists. The families of the patients also preferred endovascular treatment to open neurosurgery, as this is a less invasive treatment. Thus, off-label use of the PED was performed. All endovascular treatments were conducted by experienced neuro-interventionists. All endovascular procedures were performed under general anesthesia. After canalizing the femoral artery with a 6-F artery sheath, a 6-F guiding catheter (Codman, Raynham, Massachusetts, USA) was placed in the distal V2 segment. Marksman (EV3, Irvine, California, USA) was then navigated inside the guiding catheter to the P2 segment of the posterior cerebral artery. Once the PED reached the desired position, deployment was performed by a combination of withdrawal of the Marksman catheter and advancement of the delivery wire. If the diameter of the aneurysm or eccentric lumen exceeded 10 mm, we used the jailing technique to coil the aneurysm or eccentric lumen with the assistance of stents. Additional coiling was performed in three aneurysms through a pre-jailed Echelon-10 catheter (EV3, Plymouth, Minnesota, USA) to loosely pack the aneurysmal sac, or in vertebrobasilar junction (VBJ) aneurysms to sacrifice the contralateral vertebral artery (VA). One patient underwent parent artery occlusion using a balloon [Hyperform (EV3, Irvine, California, USA)]. If the aneurysm originated from the basilar artery with abundant perforating arteries, sole PED insertion may be considered the first choice to avoid the occurrence of ischemic events.

### Antiplatelet Treatment and Anticoagulation

All patients were premedicated with a dual antiplatelet regimen (1 mg/kg of clopidogrel and 100 mg of aspirin daily) for 5 days prior to treatment. During the procedure, an intravenous bolus dose of heparin (100 IU/kg) was administered, and heparinization was continued to maintain an activated clotting time of 2–3 times the baseline value throughout the procedure. Dual antiplatelet therapy was continued for 6 months after the procedure, and aspirin was continued for life, as per the standard embolism prophylaxis for intraluminal stents.

## Results

We evaluated the patients' pre- and post-operative clinical statuses using the modified Rankin scores (mRS), and evaluated the lesions after the procedure by follow-up digital subtraction angiography (DSA) and magnetic resonance imaging (MRI). One patient (25%) died from brainstem infarction or compression of the brainstem 3 days after the procedure. The other three patients (75%) underwent clinical and imaging follow-up 6 months after the procedure. Initial clinical and radiographic data are summarized in Table 1, while follow-up data are summarized in Table 2.

**Table 2:** Angiographic and clinical follow-up data.

**Case**	**Clinical follow-up**		**F/U period(months)**	**Angiography F/U**	**MRI F/U of Aneurysms (mm)**	
	**Pre-operational mRS**	**F/U mRS**			**D_**L**_ 1**	**D_**L**_ 2**
1	1	0	6	Well reconstruction of PA and complete occlusion of aneurysm	25	20
2	1	0	6	Well reconstruction of PA and complete occlusion of aneurysm	28	20
3	2	0	6	Complete occlusion of PA and aneurysm	26	25
4	1	6	0	–	28	–

*PA, parent artery; DL 1, largest diameter of the preoperative aneurysms; DL 2, largest diameter of the postoperative aneurysms*.

### Clinical Follow-Up

The pre- and post-operative mRS showed that the clinical presentations of three patients (75%) (cases 1, 2, and 3) achieved excellent improvement. However, the patient in case 4 died from brainstem infarction or compression of the brainstem 3 days after the insertion of four PEDs.

### Digital Subtraction Angiography Follow-Up

DSA performed 6 months after the PED procedure showed that two patients (66.7%) achieved favorable reconstruction of the parent vessel. Complete occlusion of the parent artery was observed in one patient (33.3%) who had undergone reconstructive endovascular treatment by two PEDs.

### Magnetic Resonance Imaging Follow-Up

The comparison of MRI performed 6 months after the PED procedure with preoperative MRI showed reduction of the mass effect in three patients (75%), especially in case 3.

### Case Details

#### Case 1

**Figure 1:**
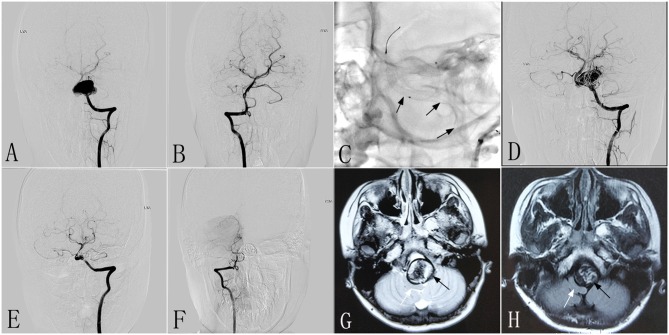
Images from a 10- to 12-year-old patient with a left vertebral dissecting aneurysm (case 1). **(A)** Preoperative anteroposterior angiogram showing a giant dissecting aneurysm in the LVA. **(B)** Preoperative anteroposterior angiogram demonstrating the patency of the RVA. **(C)** Intraprocedural unsubtracted view showing the location of the PED (4.5 × 35 mm). **(D)** Immediately postoperative angiogram of the LVA demonstrating the reconstruction of the parent vessel, and contrast stasis in the lumen of the aneurysm. **(E,F)** Six-month post-treatment anteroposterior angiograms showing the patency of the LVA **(E)** and the complete occlusion of the RVA **(F)**. **(G,H)** Six-month post-treatment MRI **(H)** compared with preoperative axial MRI **(G)** demonstrating reduction of the aneurysm size (black arrow) and increased space around the brainstem (white arrow). LVA, left vertebral artery; RVA, right vertebral artery; PED, pipeline embolization device.

A 10–12-year-old patient had experienced headaches and left-sided occipital pain for 3 months without other neurological symptoms. Computed tomographic angiography (CTA) performed at another hospital showed a giant left vertebral dissecting aneurysm. The patient was then transferred to our center. DSA showed a left vertebral dissecting aneurysm that measured 25 × 19 mm (Figures 1A,B), and MRI revealed a severe mass effect. Due to the complexity of the aneurysm, the decision was made to perform endovascular therapy with the PED. The patient had a mRS of 1, and underwent intervention therapy with a PED (4.5 × 35 mm) and coils (Figure 1C). The procedure was successful, with no complications. Immediately postoperative angiography showed that the aneurysm was incompletely occluded, with patency of the parent vessel (Figure 1D). After the procedure, the clinical symptoms of patient were mildly improved compared with preoperatively. At 6 months post-treatment, follow-up DSA demonstrated complete aneurysmal occlusion and occlusion of the right VA (RVA) (Figures 1E,F). Compared with preoperative MRI (Figure 1G), follow-up MRI (Figure 1H) showed a reduction of the mass effect and increased space of the brainstem. At 6 months after the procedure, the patient was neurologically normal, with a mRS of 0.

#### Case 2

**Figure 2:**
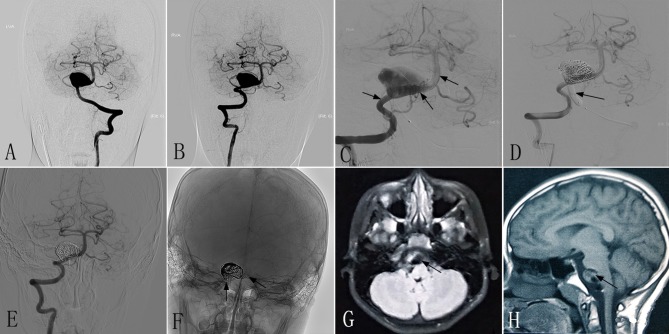
Images from an 8- to 10-year-old patient with a giant dissecting aneurysm located in the VBJ (case 2). **(A,B)** Preoperative anteroposterior angiograms of the LVA **(A)** and RVA **(B)** showing a giant dissecting aneurysm in the VBJ. **(C)** Intraprocedural unsubtracted view showing the successful insertion of PEDs (3.5 × 35 mm) (black arrow). **(D)** Intraprocedural angiogram showing complete occlusion of the LVA by a balloon (black arrow). **(E,F)** Six-month postoperative angiograms showing the reconstruction of the RVA **(E)** and stable PEDs **(F)** (black arrow). **(G,H)** Six-month postoperative MRI showing a mass effect with a diameter of 20 × 15 mm (black arrow). VBJ, vertebrobasilar junction; LVA, left vertebral artery; RVA, right vertebral artery; PED, pipeline embolization device.

An 8–10-year-old patient had experienced headaches and diplopia for 3 weeks. At another hospital, DSA showed a giant dissecting aneurysm located in the VBJ (Figures 2A,B), and MRI demonstrated a conspicuous mass effect with a diameter of 28 × 18 mm. The patient had a mRS of 1, and was transferred to our center for endovascular therapy with the PED and coils. The RVA underwent endovascular treatment with two PEDs (3.5 × 35 mm) (Figure 2C), while the left VA (LVA) underwent endovascular treatment with distal balloon occlusion (Figure 2D). Immediately postoperative angiography showed that the PEDs were inserted successfully. After the procedure, the headache was mildly improved compared with preoperatively. At 6 months post-treatment, follow-up DSA revealed that the RVA had achieved excellent reconstruction (Figures 2E,F), and the LVA was completely occluded. Follow-up MRI showed a reduction of the mass effect to a diameter of 20 × 15 mm (Figures 2G,H). The patient made an excellent recovery, with a mRS of 0.

#### Case 3

**Figure 3:**
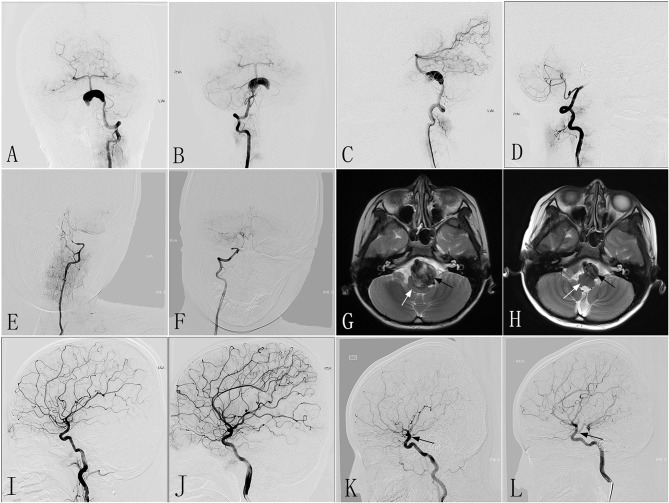
Images from an 8- to 10-year-old patient with a giant dissecting aneurysm located in the VBJ (case 3). **(A,B)** Preoperative anteroposterior angiograms of the LVA **(A)** and RVA **(B)** showing a giant dissecting aneurysm in the VBJ. **(C,D)** Immediately postoperative lateral angiograms showing the successful reconstruction of the LVA **(C)** and the complete occlusion of the RVA by coiling **(D)**. **(E,F)** Six-month post-treatment angiograms confirming the complete occlusion of the LVA **(E)** and the RVA **(F)**. **(G,H)** Six-month post-treatment MRI **(H)** compared with MRI performed 1 day post-treatment **(G)** demonstrating the reduction in the aneurysm size (black arrow) and increased space around the brainstem (white arrow). **(I,J)** Preoperative angiograms of the left and right ICA demonstrating weak dual-sided PCoAs. **(K,L)** Six-month post-treatment angiograms of the left and right ICA confirming robust dual-sided PCoAs (black arrow) providing sufficient blood for posterior circulation. VBJ, vertebrobasilar junction; LVA, left vertebral artery; RVA, right vertebral artery; ICA, internal carotid artery; PCoAs, posterior communicating arteries.

An 8–10-year-old patient with a mRS of 2 experienced a sudden onset of headaches accompanied by dysphagia 2 months before being admitted to our hospital. CTA performed in another hospital revealed a giant dissecting aneurysm located in the VBJ, which was confirmed on DSA performed in our hospital (Figures 3A,B). The LVA was treated with two PEDs (3.5 × 35 mm), and the RVA underwent parent artery occlusion with coils. Immediately postoperative angiography showed excellent reconstruction of the LVA (Figure 3C), and complete occlusion of the RVA (Figure 3D). After the procedure, the clinical symptoms were mildly improved compared with preoperatively. One day post-treatment, MRI demonstrated a giant mass effect with an intramural hematoma, resulting in severe brainstem compression. Six months post-treatment, follow-up DSA revealed complete occlusion of the LVA and RVA (Figures 3E,F). Compared with MRI performed at 1 day post-treatment (Figure 3G), follow-up MRI showed a marked reduction in the mass effect (Figure 3H). At 6 months after the procedure, the patient had no clinical problems and/or focal neurological function deficiency, with a mRS of 0. As this patient had weak dual posterior communicating arteries preoperatively (Figures 3I,J), the good clinical outcome might be attributed to the presence of robust dual posterior communicating arteries after the procedure (Figures 3K,L).

#### Case 4

**Figure 4:**
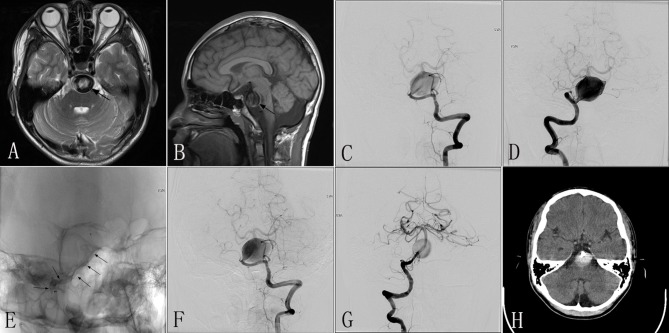
Images from a 10- to 12-year-old patient with a giant dissecting aneurysm located in the basilar artery (case 4). **(A,B)** Preoperative MRI showing a basilar giant dissecting aneurysm with compression of the brainstem (black arrow). **(C,D)** Preoperative anteroposterior angiograms of the LVA **(C)** and RVA **(D)** confirming a giant dissecting aneurysm located in the basilar artery. **(E)** Intraprocedural unsubtracted view showing successful insertion of the PED (3.5 × 35 mm) (black arrow). **(F,G)** Immediately postoperative anteroposterior angiograms of the LVA **(F)** and RVA **(G)** demonstrating excellent reconstruction of the basilar artery and evident contrast stasis in the lumen of the aneurysm. **(H)** CT performed 1 day after the procedure confirming no indications of SAH. LVA, left vertebral artery; RVA, right vertebral artery; PED, pipeline embolization device; SAH, subarachnoid hematoma.

A 10–12-year-old patient experienced chronic headaches and vertigo for 8 months. MRI performed in another hospital demonstrated a giant mass effect with an intramural hematoma causing severe brainstem compression (Figures 4A,B). DSA revealed a giant dissecting aneurysm located in the basilar artery (Figures 4C,D). The patient was then transferred to our center. Due to the large size of this lesion, four PEDs (3.5 × 35 mm) were inserted to repair the wall of the basilar artery (Figure 4E). The patient tolerated the procedure well. Immediately postoperative angiography showed that the lumen of the aneurysm had evident contrast stasis with the patency of the parent artery (Figures 4F,G). One day post-treatment, the patient experienced an acute onset of dysphasia and right hemiplegia; computed tomography (CT) was immediately performed and did not show any significant findings (Figure 4H). The post-procedural symptoms were thought be related to contrast neurotoxicity due to the large dosage of contrast agent administered during the procedure, and so we did not perform repeat MRI. The symptoms were gradually alleviated by rehydration therapy and symptomatic treatment. At 3 days after the procedure, the patient had an acute onset of headache and dizziness with loss of consciousness. While on the way to undergo repeat CT, the patient became apneic. The patient was immediately transferred to the Neurosurgical Intensive Care Unit, and underwent positive rescue treatments of airway protection; however, the patient was unable to recover the ability to self-breathe, lost consciousness, and died.

## Discussion

The PED technique is a new treatment modality for intracranial aneurysms, particularly giant aneurysms, for which an excellent outcome cannot be achieved via conditional endovascular treatment (6). Flow diversion is a new and relatively untested technology in children, although the outcomes in adults are promising. For challenging lesions in the pediatric population, flow diversion may have a valuable role as a well-tolerated, safe treatment with durable results (1). To date, few studies have confirmed the safety and effectiveness of the PED technique in the treatment of pediatric giant dissecting aneurysms located in the vertebrobasilar system, and most of the relevant literature is comprised of case reports. Additionally, most published case reports only describe successful examples, which may give misleading information on the outcome of the PED technique. To objectively consider the feasibility and effectiveness of the PED technique in this population, we described our early experience with the PED technique in four consecutive pediatric patients.

The management of VBDAs is technically challenging due to their morphologic features and localization (7). Giant vertebrobasilar aneurysms carry a high rate of morbidity, and no treatment modality has yet significantly improved the dismal natural history of the lesion (8). Furthermore, the treatment of pediatric giant VBDAs is more complex, and there is minimal literature reporting the management of this lesion. It is uncommon for a dissecting aneurysm to resolve spontaneously or with medical treatment only (9). Hence, we need to prevent the progression of these lesions, particularly in pediatric patients who have longer expected lifespans than adults.

Microsurgical clipping of the aneurysm is the most effective method for obliterating the aneurysm, and carries the lowest risk of recurrence (10). However, there are two major limitations of this method for dissecting aneurysms located in vertebrobasilar arteries. First, the aneurysm neck cannot be identified for clipping, and the wall friability can make surgical clipping difficult and risky (9). Second, the anatomic locations of these lesions adjacent to the brainstem increase the difficulty of the surgical treatment, with higher risks of mortality and morbidity (10). Thus, endovascular techniques are preferred for these lesions (11–13). However, for these particular lesions, traditional endovascular treatments (such coiling, stent-assisted coiling, and balloon-assisted coiling) result in a high recurrence rate (14). Additionally, pediatric patients can tolerate deconstructive treatment better than adults because of a greater functional brain capacity and a better compensatory blood supply (15–17). In the majority of reported cases in the pediatric population, the chosen treatment for dissecting, fusiform, and giant partially thrombosed aneurysms was parent vessel sacrifice (either surgically or endovascularly), with good clinical results (18–20). However, in situations in which parent artery preservation is mandatory given the expected long life spans of children and the branches of the vessels, the use of stent-assisted techniques may be the most appropriate choice (21). Thus, endovascular treatment via the insertion of a PED may be a viable alternative.

Currently, a significant proportion of intracranial aneurysms in adults are successfully treated with flow diverters. Flow diverters have also emerged as an effective and safe alternative in small case series, which report favorable outcomes in young children (17), particularly for VBDAs. To the best of our knowledge, there have only been seven reported cases (seven aneurysms) of PED insertion in children with VBDAs, including four giant aneurysms, two large aneurysms, and one small aneurysm (6, 17, 22–25) (Table 3). The literature indicates that the use of flow diverters in children is considered positive, particularly for VBDAs; hence, the treatment modalities for these lesions have gradually shifted from parent artery occlusion to the PED technique. Similar to the current literature, we report acceptable therapeutic outcomes for these complex lesions; although one patient died from brainstem compression or infarction, the other patients were able to resume normal life without major neurological deficiency. Additionally, it was crucial to determine whether the mass effect resulting from these complex lesions could be alleviated compared with the pretreatment status. We confirmed that the mass effect in the three surviving patients was reduced on follow-up MRI, in accordance with previous case reports (17, 22, 23, 26).

**Table 3:** Literature summary of the use of the PED technique for pediatric vertebrobasilar dissecting aneurysms.

**References**	**Age (years)/Sex**	**Type of lesion**	**Location**	Size (mm)^*^	**Status**	**Number of devices used**	**Adjunctive coiling**	**Complete aneurysm occlusion at last imaging follow up**	**Good clinical outcome^**&**^**
Fiorella et al. (25)	13/F	Dissecting	Basilar artery	39	Unruptured	7	No	Yes	Yes
D'Urso et al. (6)	12/F	Dissecting	Basilar artery	Giant	Ruptured	2	N/A	No	Yes
	5/F	Dissecting	Basilar tip	Small	Ruptured	2	N/A	Yes	No
	7/M	Dissecting	Vertebrobasilar junction	Giant	N/A	4	N/A	Yes	Yes
Anna et al. (22)	15/M	Dissecting	RVA	19.5	Unruptured	2	No	Yes	Yes
Vargas et al. (24)	9/M	Dissecting	Basilar artery	20	Unruptured	2	No	No	Yes
Mohammad et al. (23)	15/M	Dissecting	Basilar artery	39	Unruptured	1	Yes	Yes	Yes

* *Aneurysm size was not specified in most studies*.

& *The definition of “good clinical outcome” varied between manuscripts, but overall consisted of a modified Rankin score of 0–1, Glasgow Outcome Scale of 4–5, or patients who were asymptomatic at the last clinical follow up and had no significant neurological impairment. F, female; M, male; N/A, no data available*.

Although the PED technique was approved by the Food and Drug Administration in 2011 for the treatment of large or giant wide-necked anterior circulation aneurysms in the internal carotid artery from the petrous to the superior hypophyseal segments in adults (27), the use of the PED technique for pediatric dissecting aneurysms was off-label. This approach was applied to this particular population after a multidisciplinary discussion, under the condition that conservative therapeutic methods could not achieve a good outcome. Furthermore, from a morphometric standpoint, we found that the size range of currently available intracranial stents or flow diverters is sufficient to cover the pediatric population, and intracranial arterial diameters in children do not undergo extensive growth, especially after early childhood (28). Hence, endovascular treatment with PEDs may become a feasible choice for pediatric complex lesions, such as giant VBDAs.

In our case series, follow-up DSA showed that case 3 had complete occlusion of the LVA at 6 months post-treatment. We consider that the cause of this parent vessel complete occlusion might have been an in-stent thrombus. Thus, strict attention must be paid to pre- or post-procedural antiplatelet medication protocols for flow diverters. There is no standard antiplatelet/anticoagulant therapy for children undergoing intracranial placement of vascular scaffolds (such as stents, stent grafts, or flow diverters) (28), and antiplatelet administration for endovascular treatment is extremely variable (29–31). Furthermore, the antiplatelet/anticoagulant therapy for adults undergoing PED insertion for aneurysms in the posterior circulation is also variable (32–34). In children, a weight-based dose of 0.2–1 mg/kg/day is associated with a 43% platelet aggregation inhibition response. However, weight-based dose calculations extrapolated from an adult dosage of 75 mg of clopidogrel per day are not only misleading, but may also lead to life-threatening consequences (35, 36). Some previously reported cases experienced thrombotic complications and hemorrhagic events because of inappropriate antiplatelet therapy (24, 37, 38). It is important to be aware of the different sensitivity of each child to the dual antiplatelet regimen. Furthermore, there is a need for age-specific reference ranges (39). In this particular population, initiating appropriate antiplatelet therapy before and after treatment could minimize the risks of hemorrhage and ischemic events as a result of parent artery occlusion and in-stent stenosis.

The patient in case 4 died 3 days after the procedure, although this patient tolerated the procedure well and CT showed no signs of subarachnoid hematoma. Multidisciplinary discussion by many experts attributed the death to two potential causes: severe compression of the brainstem due to acute thrombosis, or brainstem infarction associated with acute thrombosis in the stent. The parents of the patient did not consent to an autopsy to confirm the cause of death. A risk-benefit evaluation must be considered before selecting a treatment modality for pediatric patients with a severe mass effect or large intracranial hematoma. Although the parents of the patient in case 4 refused a surgical operation, surgical treatment with an artery bypass may have been an alternative method. A multidisciplinary approach to managing aneurysms facilitates the attainment of good outcomes in this diverse and challenging group of patients.

### Limitations

As pediatric giant VBDAs are rare, the present study was only able to include four cases. Additionally, although long-term follow-up is important in this special population that appears to be at greater risk of delayed complications, follow-up was limited to an average of 6 months. Moreover, we did not check the aspirin and Plavix response before and after procedure, which is an important measure to reduce the risk of ischemic and/or hemorrhagic events. Finally, there was a lack of suitable controlled patients treated via other therapeutic methods. A prospective, multicenter investigation with a large sample is essential.

## Conclusions

PEDs could be feasible in the treatment of pediatric giant VBDAs. However, the safety and efficacy of this method have not been clarified in this special population, and long-term follow-up is still necessary.

## Author Contributions

JW and YZ performed the manuscript writing. ZT, LJ, WZ, WL, JC, XD, ZW, and KW acquired the data. JL, PL, ZM, and YZ analyzed and interpreted the data. ML and XY conceived and designed the research, and handled funding and supervision.

### Conflict of Interest Statement

The authors declare that the research was conducted in the absence of any commercial or financial relationships that could be construed as a potential conflict of interest.
