# Corrigendum: Application of the Pipeline Embolization Device for Giant Vertebrobasilar Dissecting Aneurysms in Pediatric Patients

**DOI:** 10.3389/fneur.2019.00862

**Published:** 2019-08-22

**Authors:** Jiejun Wang, Yisen Zhang, Ming Lv, Xinjian Yang, Zhongbin Tian, Jian Liu, Peng Liu, Zefeng Miao, Luqiong Jia, Junfan Chen, Xinghuan Ding, Ying Zhang, Wei Zhu, Wenqiang Li, Kun Wang, Zhongxiao Wang

**Affiliations:** ^1^Beijing Neurosurgical Institute, Beijing Tiantan Hospital, Capital Medical University, Beijing, China; ^2^Heping Hospital Affiliated to Changzhi Medical College, Changzhi, China

**Keywords:** pediatric, dissecting, giant, vertebrobasilar, pipeline

In the original article, Cunegatto-Braga et al., 2018 was not cited in the article. The citation has now been inserted in the ***Discussion***, paragraph four, and should read:

“Currently, a significant proportion of intracranial aneurysms in adults are successfully treated with flow diverters. Flow diverters have also emerged as an effective and safe alternative in small case series, which report favorable outcomes in young children (17), particularly for VBDAs. To the best of our knowledge, there have only been seven reported cases (seven aneurysms) of PED insertion in children with VBDAs, including four giant aneurysms, two large aneurysms, and one small aneurysm (6, 17, 22–25) (Table 3). The literature indicates that the use of flow diverters in children is considered positive, particularly for VBDAs; hence, the treatment modalities for these lesions have gradually shifted from parent artery occlusion to the PED technique. Similar to the current literature, we report acceptable therapeutic outcomes for these complex lesions; although one patient died from brainstem compression or infarction, the other patients were able to resume normal life without major neurological deficiency. Additionally, it was crucial to determine whether the mass effect resulting from these complex lesions could be alleviated compared with the pretreatment status. We confirmed that the mass effect in the three surviving patients was reduced on follow-up MRI, in accordance with previous case reports (17, 22, 23, 26).”

We apologize for this error and state that this does not change the scientific conclusions of the article in any way. The original article has been updated.

## References

[17] Cunegatto-Braga M Hogan B Aguilar-Salinas P Beier AD Hanel RA. Pipeline embolization device flow diversion for a dissecting ruptured posterior cerebral artery aneurysm in a pediatric patient. World Neurosurg. (2018) 117:255–60. 10.1016/j.wneu.2018.06.031 29909213

